# Expression Profile of New Gene Markers Involved in Differentiation of Canine Adipose-Derived Stem Cells into Chondrocytes

**DOI:** 10.3390/genes13091664

**Published:** 2022-09-16

**Authors:** Maurycy Jankowski, Mariusz Kaczmarek, Grzegorz Wąsiatycz, Aneta Konwerska, Claudia Dompe, Dorota Bukowska, Paweł Antosik, Paul Mozdziak, Bartosz Kempisty

**Affiliations:** 1 Department of Anatomy, Poznan University of Medical Sciences, 60-701 Poznan, Poland; 2 Doctoral School, Poznan University of Medical Sciences, 60-701 Poznan, Poland; 3 Department of Cancer Immunology, Chair of Medical Biotechnology, Poznan University of Medical Sciences, 61-866 Poznan, Poland; 4 Gene Therapy Laboratory, Department of Cancer Diagnostics and Immunology, Greater Poland Cancer Centre, 61-866 Poznan, Poland; 5 Department of Veterinary Surgery, Institute of Veterinary Medicine, Nicolaus Copernicus University in Torun, 87-100 Torun, Poland; 6 Department of Histology and Embryology, Poznan University of Medical Sciences, 60-701 Poznan, Poland; 7 Center for Advanced Technology, Department of Molecular and Cellular Biology, Institute of Molecular Biology and Biotechnology, Faculty of Biology, Adam Mickiewicz University, 61-614 Poznan, Poland; 8 Department of Diagnostics and Clinical Sciences, Institute of Veterinary Medicine, Nicolaus Copernicus University in Torun, 87-100 Torun, Poland; 9 Prestage Department of Poultry Science, North Carolina State University, Raleigh, NC 27695, USA; 10 Department of Obstetrics and Gynecology, University Hospital and Masaryk University, 601 77 Brno, Czech Republic

**Keywords:** adipose, stem cells, chondrocytes, differentiation, RNAseq, transcriptomics

## Abstract

The interest in stem cell research continuously increased over the last decades, becoming one of the most important trends in the 21st century medicine. Stem cell-based therapies have a potential to become a solution for a range of currently untreatable diseases, such as spinal cord injuries, type I diabetes, Parkinson’s disease, heart disease, stroke, and osteoarthritis. Hence, this study, based on canine material, aims to investigate the molecular basis of adipose-derived stem cell (ASC) differentiation into chondrocytes, to serve as a transcriptomic reference for further research aiming to introduce ASC into treatment of bone and cartilage related diseases, such as osteoarthritis in veterinary medicine. Adipose tissue samples were harvested from a canine specimen subjected to a routine ovariohysterecromy procedure at an associated veterinary clinic. The material was treated for ASC isolation and chondrogenic differentiation. RNA samples were isolated at day 1 of culture, day 30 of culture in unsupplemented culture media, and day 30 of culture in chondrogenic differentiation media. The resulting RNA was analyzed using RNAseq assays, with the results validated by RT-qPCR. Between differentiated chondrocytes, early and late cultures, most up- and down-regulated genes in each comparison were selected for further analysis., there are several genes (e.g., *MMP12*, *MPEG1*, *CHI3L1*, and *CD36*) that could be identified as new markers of chondrogenesis and the influence of long-term culture conditions on ASCs. The results of the study prove the usefulness of the in vitro culture model, providing further molecular insight into the processes associated with ASC culture and differentiation. Furthermore, the knowledge obtained could be used as a molecular reference for future in vivo and clinical studies.

## 1. Introduction

Over the last several decades, the interest in stem cell research continuously increased, making it one of the most important trends of the 21st century medicine. There is an agreement in the scientific community that stem cell-based therapies have a significant potential to become a solution for a range of currently untreatable diseases, such as spinal cord injuries, type I diabetes, Parkinson’s disease, heart disease, stroke, and osteoarthritis [1,2,3,4,5,6]. Nonetheless, while there are already some stem cell-based therapeutic approaches, a significant amount of information about their properties, most notably the molecular mechanisms governing their function, plasticity, and differentiation, remain undiscovered. This sparks concerns about the applicability of various stem cell types in a clinical setting, mostly regarding the efficiency and potential side effects of their use [7,8]. Hence, there is a significant need for complex studies analyzing the molecular basis of stem cell function, to fully understand their potential and safety in possible therapeutic applications [9].

There are multiple sources of stem cells, including embryonic and adult stem cells. While embryonic stem cells are characterized by a significant differentiation potential and plasticity, they are relatively hard to obtain in appropriate amounts. Hence, adult stem cells, such as bone marrow stem cells (BMSCs) are the source used for most of the currently developed therapeutic approaches [10,11]. Moreover, there have recently been some significant developments regarding another source of adult mesenchymal stem cells (MSCs), namely adipose-derived stem cells (ASCs) [12,13,14,15,16]. These stem cells can be freely obtained from the adipose tissue of adult mammals, and exhibit very similar characteristics to BMSCs, while being much easier to collect without almost any patient burden. Furthermore, ASCs have been proven to be able to differentiate into a range of different lineages, including osteoblasts, chondrocytes, adipocytes and neural cells, making them a versatile candidate for a range of therapies, mostly related to the field of regenerative medicine [9,17].

Using a model based on *Canis familiaris* (dog) material, we aimed to investigate the molecular basis of ASC differentiation into chondrocytes, to serve as a transcriptomic reference for further research aiming to introduce ASC into treatment of cartilage related veterinary diseases, such as osteoarthritis. The canine model was chosen due to the wide availability of dog adipose tissue, a remnant material of routine surgeries, as well as the possibility of use of the obtained results as a reference for further in vivo and clinical studied in the field of veterinary medicine [18,19]. Furthermore, there is an added potential of future cross-referencing of the knowledge obtained with similar datasets regarding other experimental models, such as mice, or even humans. Hence, this study aimed to examine the genes of most significantly altered expression genes after chondrogenic differentiation of canine ASCs, to evaluate the effects of induced lineage progression and prolonged ex vivo environment on the identity of ASCs and differentiated chondrocytes.

## 2. Material and Methods

### 2.1. Material Collection

Small (<1 cm^3^) samples of adipose tissue, were collected from *c. familiaris* specimens subjected to a routine ovariohysterectomy procedure at a commercial veterinary clinic. The material used in this study is usually considered waste and discarded following surgery. Hence, as it does not require any additional surgical procedures, the study was exempt from local bioethical committee approval. Following collection, the samples were transported to the laboratory in Dulbecco’s phosphate-buffered saline (DPBS; Sigma-Aldrich, Saint Louis, MO, USA), supplemented with 1% of antibiotic antimycotic solution (A5955, Sigma-Aldrich, Saint Louis, MO, USA), and processed no longer than 24 h after collection.

### 2.2. Cell Sample Preparation

In the first stem, a double washing of the adipose tissue samples in ice cold PBS was performed to remove any remnant blood. The tissue was minced until homogenous with the use of sterile surgical blades in a Petri dish. Next, the samples enzymatically digested using a 1 mg/mL Type I collagenase solution (Gibco, Thermo-Fischer Scientific, Waltham, MA, USA) for 40 min at 37 °C. The digestion tubes were vortexed every 10 min during the incubation period. Afterwards, the enzyme activity was inhibited with the use of 1 mL of fetal bovine serum (FBS, Sigma-Aldrich, Saint Louis, MO, USA), and the samples were centrifuged at 1200× *g* for 10 min. Supernatant liquid from above the resulting cell pellet was discarded, after which the pellet was resuspended in DPBS and centrifuged again for 10 min at 500× *g*. Following supernatant removal, the cells were resuspended in 4 mL of Dulbecco’s Modified Eagle’s Medium—high glucose (DMEM, Sigma-Aldrich, Saint Louis, MO, USA), supplemented with 10% FBS (Sigma-Aldrich, Saint Louis, MO, USA), 4 mM of L-glutamine (Sigma-Aldrich, Saint Louis, MO, USA) and 1× antibiotic–antimycotic solution (A5955, Sigma-Aldrich, Saint Louis, MO, USA). The resulting cell solution was placed in a 25 cm^2^ cell culture flasks (two flasks for each canine specimen, to provide early controls without further disturbance of differentiation samples).

### 2.3. Flow Cytometry Analysis

Before the culture, samples of cells suspended in the culture medium were collected for antibody staining and flow cytometry analysis. The surface markers required for ASC identification were chosen based on the minimal criteria presented by Dominici et al., as well as the systematic review of Mildmay-White et al. [20,21]. As not all of the proposed canine ASC marker and isotype control antibodies were commercially available, and due to a large number of cell samples screened and the significant cost of the individual antibodies, a panel of four antibodies (two positive and two negative selected from those most commonly described in literature regarding ASCs) was applied to initially assess the identity of the studied cells, with further morphological and differentiation analyses serving as a final confirmation. Hence, the staining was performed using the following antibodies: rat anti-dog CD44: FITC (11-5440-41, Thermo-Fischer Scientific, Waltham, MA, USA), rat anti-dog CD90: PE (12-5900-41, Thermo-Fischer Scientific, Waltham, MA, USA), rat anti-dog CD45: APC (MCA1042APC, Bio-Rad, Hercules, CA, USA) and mouse anti-dog CD34: Alexa Fluor^®^ 647 (MCA2411A647, Bio-Rad, Hercules, CA, USA), as well as respective isotype controls: rat IgG2ak: FITC (11-5440-41, Thermo-Fischer Scientific, Waltham, MA, USA), rat IgG2bk: PE (12-4031-81, Thermo-Fischer Scientific, Waltham, MA, USA), rat IgG2b: APC (MCA6006APC, Bio-Rad, Hercules, CA, USA) and mouse IgG1: Alexa Fluor^®^ 647 (MCA928A647, Bio-Rad, Hercules, CA, USA). 1:50 dilution was used for all the antibodies and isotype controls, with the staining process conducted according to manufacturer protocols. The stained samples were analyzed using the BD FACSAria™ cytometer (Becton Dickinson, Franklin Lanes, NJ, USA).

### 2.4. In Vitro Cell Culture

Initially, the in vitro cultures (IVC) were maintained for 3 days, or until ~90% confluency was observed, with the exception of early control samples, from which RNA was isolated at day 1 of culture. Next, the cells were detached from culture flasks with the use of 1× Trypsin solution (Sigma-Aldrich, Saint Louis, MO, USA) and moved to 96-well U-bottom plates. Subsequently, the cells formed spheroids and were cultured in DMEM until the 30th day of culture, with a medium change every 72 h. Overall, the study material was isolated from cell cultures at three time periods: 1st day of culture, 30th day of culture without chondrogenic differentiation and from differentiated chondrocytes. The cultured spheroids were photographed using an inverted light microscope (Ixplore Standard, Olympus, Tokyo, Japan) every 24 h to assess the potential changes in their size and morphology.

### 2.5. Chondrogenic Differentiation

For differentiation, the medium in the 96-well U-bottom plates containing ASC spheroids was exchanged to a commercially sourced chondrocyte differentiation medium (CN411D-250, Cell Applications, Inc., San Diego, CA, USA). Shortly The collected spheroids were fixed for in formaldehyde, dehydrated, embedded in paraffin blocks, and cut with a rotary microtome into sections of 3–4 µm. Obtained paraffin sections were deparaffinized in an alcohol concentration gradient, as previously described [22], stained with Alcian Blue (Sigma-Aldrich, Saint Louis, MO, USA) according to the manufacturers protocol, and reparaffinized. Analysis of all sections was performed under a light microscope, with pictures taken using the integrated camera module (Ixplore Standard, Olympus, Tokyo, Japan).

### 2.6. RNA Isolation

RNA isolation from samples was performed at three ASC culture stages: day 1 (early control), day 30 of culture in DMEM on a 96-well U-bottom plate (late culture control), and day 30 of culture in chondrogenic differentiation media (differentiated chondrocytes). The cells directed for RNA isolation were collected from cultures with medium using a serological pipette and transferred into 1mL of the TRIzol reagent (Thermo-Fischer Scientific, Waltham, MA, USA). Then, the samples were stored frozen at −80 °C. RNA isolation was conducted with the use of the TRIzol Plus Purification KIT (12183555, Thermo-Fischer Scientific, Waltham, MA, USA), as described in the manufacturer’s protocol. The evaluation of the total amount of collected RNA was conducted using optical density at 260 nm, while its purity was determined based on the 260/280 nm absorption ratio (NanoDrop 2000 spectrophotometer, Thermo-Fischer Scientific, Waltham, MA, USA). Further studies were only based on samples a 260/280 absorption ratio higher than 1.8 and RNA content over 1 mg.

### 2.7. RNAseq Analysis

The RNAseq analysis was performed by CeGaT GmbH (Tübingen, Germany) with the use of the Illumina platform (Illumina, San Diego, CA, USA). Each of the culture periods consisted of three biological repeats, with three sets of samples from day 1 (early control), day 30 (late control) and differentiated chondrocytes obtained from different animals, to mitigate the effect of inter-specimen variation. Prior to analysis the samples were subjected to additional quality control, based on the Qubit RNA (Thermo-Fischer Scientific, Waltham, MA, USA) and Bioanalyzer RNA (Aglient Technologies, Santa Clara, CA, USA). RNA integrity numbers (RINs) of the samples exhibited values between 9.1 and 10, which allowed for their further processing. 100 ng of RNA per sample was used for preparation of the cDNA library using TruSeq Stranded mRNA kit (Illumina, San Diego, CA, USA). The sequencing itself was conducted using NovaSeq 6000 (Illumina, San Diego, CA, USA). In turn, sequencing reads demultiplexing was performed using bcl2fastq (v. 2.20), with adapter trimming conducted using Skewer (v. 0.2.2) [23]. The trimmed raw reads were aligned to the CanFam3.1 canine genome with the use of STAR (v. 2.5.2b).

### 2.8. RT-qPCR Validation

Simultaneously, the RNA samples were reverse transcribed with the use of Transcriptor First Strand cDNA Synthesis Kit (Roche Life Sciences, Basel, Switzerland) and the Eppendorf Mastercycler ^®^ nexus (Eppendorf AG, Hamburg, Germany), as specified manufacturer protocols. The RT-qPCR validation was then conducted on a Lightcycler 96 (Roche Life Sciences, Basel, Switzerland) with Eva Green (Syngen Biotech, Wrocław, Poland) serving as a detection dye. The reaction mix comprised 0.5 μL cDNA, 0.5 μL forward + reverse primer mix, 2 μL of Eva Green and 7 μL of PCR-grade water. The specific primers were designed based on the transcript sequences contained in the Ensembl database [24], with the use of the Primer3 software (Table 1) [25]. Primer design accounted for the presence of all of the known protein-coding transcript variants. The 2^−ΔΔCT^ method was used to calculate the results of the analysis, with ACTB and HPRT serving as housekeeping genes [26].

### 2.9. Bioinformatical and Statistical Analysis

The number of reads assigned to specific geneIDs were compiled in the raw count files. The datasets were normalized with the use of the DESeq2 package (v. 1.24) in R (v. 4.0.3). ENTREZ gene numbers were assigned to the normalized list of genes using Bioconductor (v.3.12.0) and the org.cf.eg.db package. The ENTREZ annotated datasets were uploaded to the IDEP.91 software for processing and visualization. IDEP is an integrated web application-based simple user interface used for advanced bioinformatical analysis of RNAseq data [27].

The relationship between samples was visualized using principal component analysis (PCA). To ensure that all genes equally contribute to inter-sample distance, edgeR transformation was used to stabilize the variance across the mean [28]. Next, the gene list was filtered to extract differentially expressed gene (DEG). Fold change (FC) was calculated based on mean expression values of each gene in the three analyzed sample groups. In turn, *p* value obtained from the Wald test determined statistical significance of the results, which was corrected for multiple comparisons using the Benjamini and Hochberg’s false discovery rate. Hence, DEG selection was based on FC ≥ 2 and adjusted *p* value ≤ 0.05. A volcano plot was used to present the results of the selection, with each point signifying a gene, and with log2 of the fold change and adj. *p* value presented on the y and x axes, respectively. In this study, the 10 most upregulated and downregulated genes between the sample groups (differentiated vs. day 1; differentiated vs. day 30) were selected. Gene of interest expression was visualized using heatmaps, with sample color intensity indicating the scale of expression change between sample groups. Finally, the gene of interest lit was uploaded to the STRING software, to visualize the predicted interactions between their protein products [29].

## 3. Results

### 3.1. Flow Cytometry Analysis

**Figure 1:**
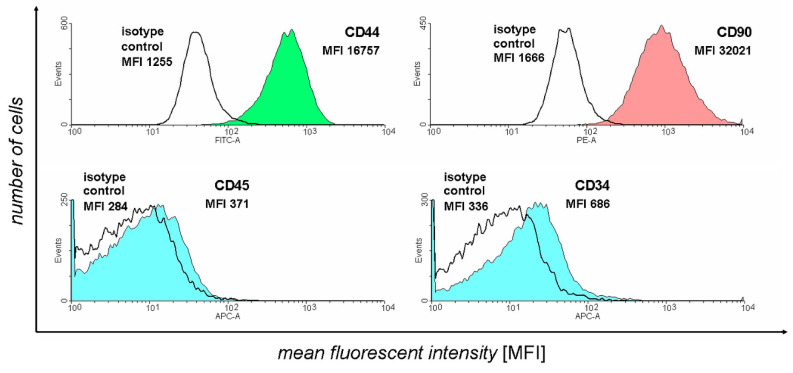
The results of flow cytometry analysis of selected ASC markers in the cell samples subjected to in vitro culture.

Flow cytometry analysis was used to confirm the identity of harvested ASCs. The results of this assay were presented on Figure 1.

The figure represents the number of acquisition events for different intensity fluorescence signals. For each antibody, a corresponding isotype control was used to background correct the fluorescence results unrelated to antibody binding. The two positive ASC markers selected (CD44 and CD90) show a large amount of detection events of larger fluorescence in comparison to isotype controls. In turn, negative marker detection results (CD45 and CD34) demonstrate that the isotype control and antibody-stained samples exhibit similar fluorescence levels, confirming the absence of their expression. Overall, the results supported the isolated cell samples’ ASC identity, with only samples with the above-mentioned marker expression pattern used for the further culture and differentiation.

### 3.2. Morphological Analysis

To assess the changes in the size and morphology of the cultured ASC spheroids, photographs of the culture wells were taken daily. The results of the initial morphological evaluation of 2D cultured cells analyzed in this study, supporting their identity and suitability for further analysis, were presented in a previous work of our team [30].

**Figure 2:**
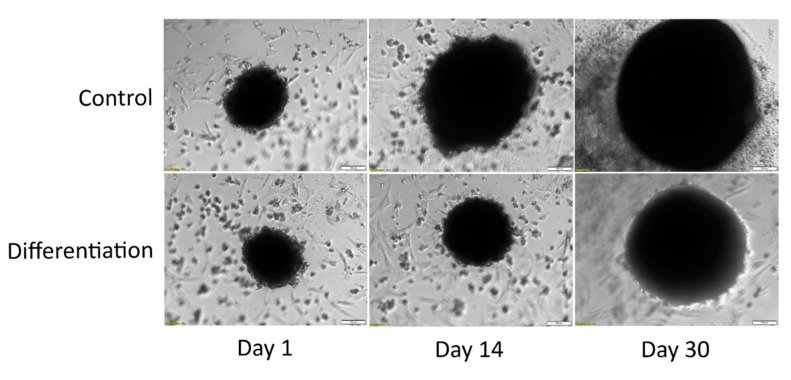
Morphological analysis results of ASC primary 3D cultures. The pictures were taken at a 10× magnification.

While the density of the cultured spheroids makes it hard to distinguish the morphological features of the constituting cells, the visual assessment allowed for a comparison of size difference on day 1, 14, and 30 of both control and differentiated culture. As can be seen on Figure 2, the spheroids subjected to unsupplemented culture exhibited a significant increase in size, visible even on its 30th day.

The differentiated chondrocyte spheroids were characterized by a significantly smaller size, with a miniscule increase on Day 14 and slightly more visible growth by day 30 of culture.

### 3.3. Evaluation of Chondrogenic Differentiation

**Figure 3:**
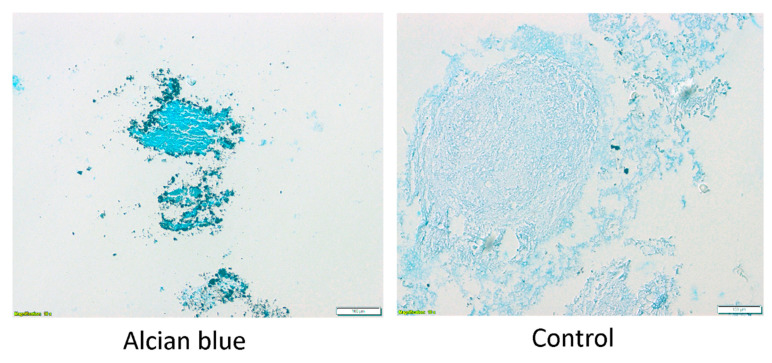
The results of alcian blue staining of chondrocyte and control spheroid slides. The pictures were taken at a 10× magnification.

To confirm the success of chondrogenic ASC differentiation, the differentiated and control spheroids were cut into slices, placed on slides, and stained with alcian blue (Figure 3). The results of osteoblast differentiation of the cells analyzed in this study, further confirming their ASC identity, were already presented in a previous work of our team [30].

The alcian blue staining confirms the ASCs differentiation into chondrocytes. Chondrocyte spheroids present deep blue staining, while control slides are only faintly colored. Furthermore, the size difference between differentiated and undifferentiated spheroid is notable, with the former presenting significantly smaller diameter. Overall, the expression of specific surface markers by the analyzed cells, their characteristic morphology, as well as their ability to differentiate into osteoblasts and chondrocytes, confirmed by specific assay results comparable to those reported in the literature, support their ASC identity with a high degree of confidence.

### 3.4. RNAseq Analysis

**Figure 4:**
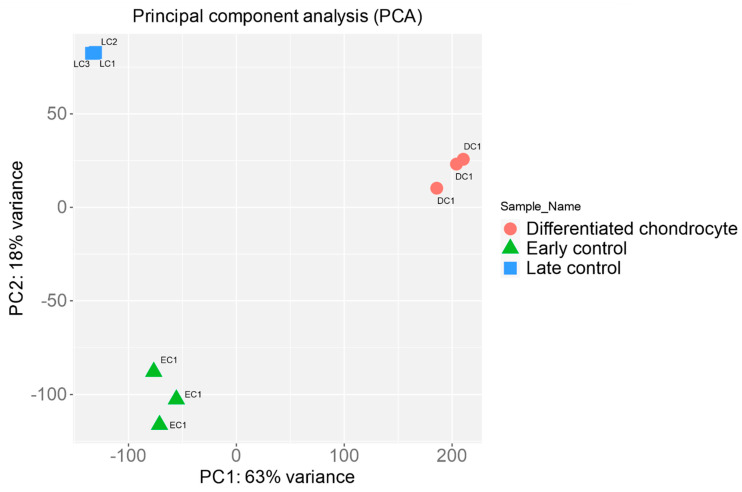
Principal component analysis of the examined control and differentiated ASCs sample groups.

Firstly, the RNAseq results were subjected to principal component analysis, to visualize the variance between the analyzed groups of samples. The results of PCA were presented in Figure 4.

The results prove that the variance inside particular sample groups is rather small, while the inter-group variance is significant. This confirms the success of chondrogenic differentiation, as well as the pronounced effects of long-term in vitro culture on ASCs.

**Figure 5:**
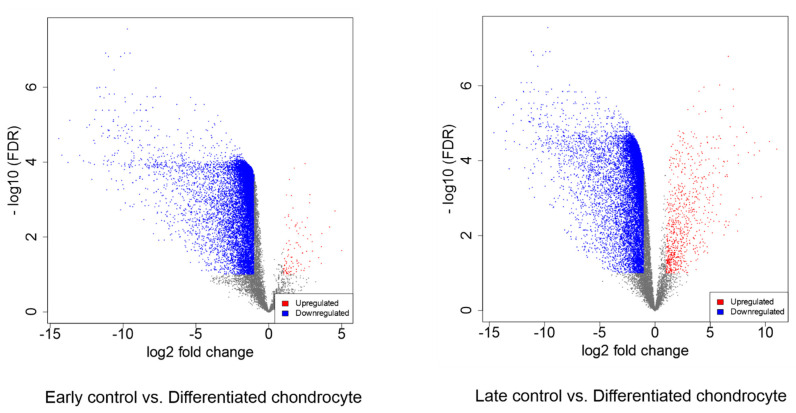
Volcano plots representing sample group composition, proportion, and distribution of differentially expressed genes.

In the further analyses, differentially expressed genes were selected (fold change > |2|, adj. *p* value < 0.05). The initial results of this selection were presented as volcano plots in Figure 5.

**Figure 6:**
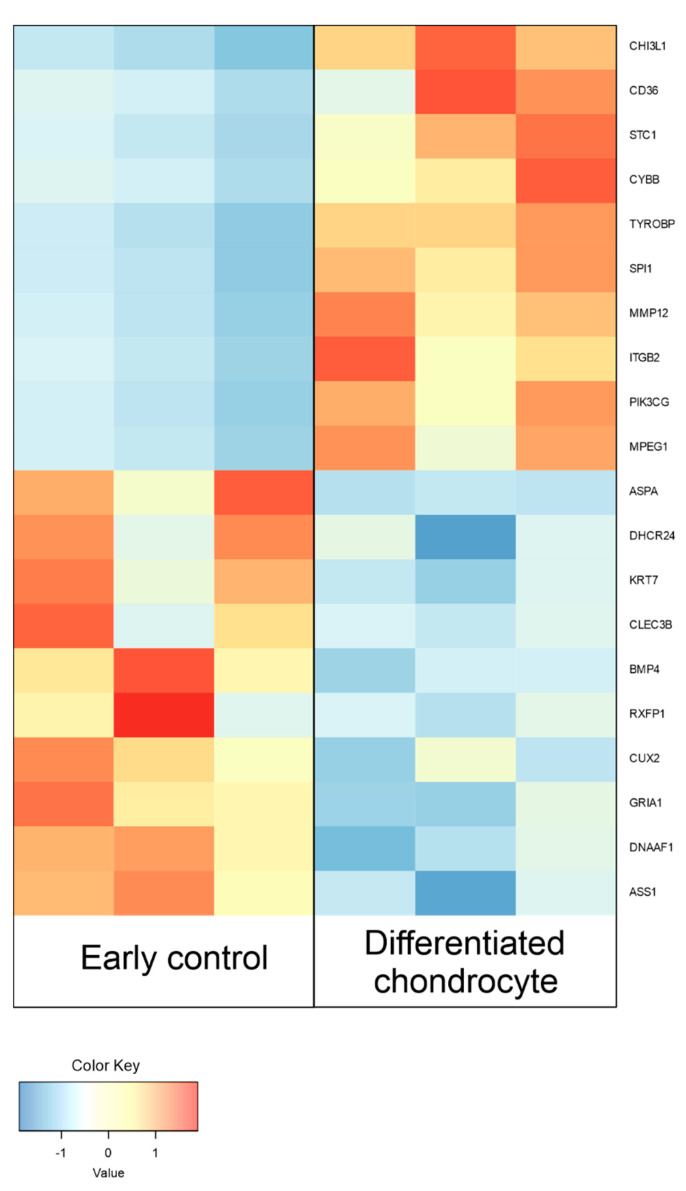
Heatmap presenting the changes in the 10 most up- and down-regulated genes between differentiated chondrocytes and day 1 primary ASC culture (early control), presented as log2FC.

**Figure 7:**
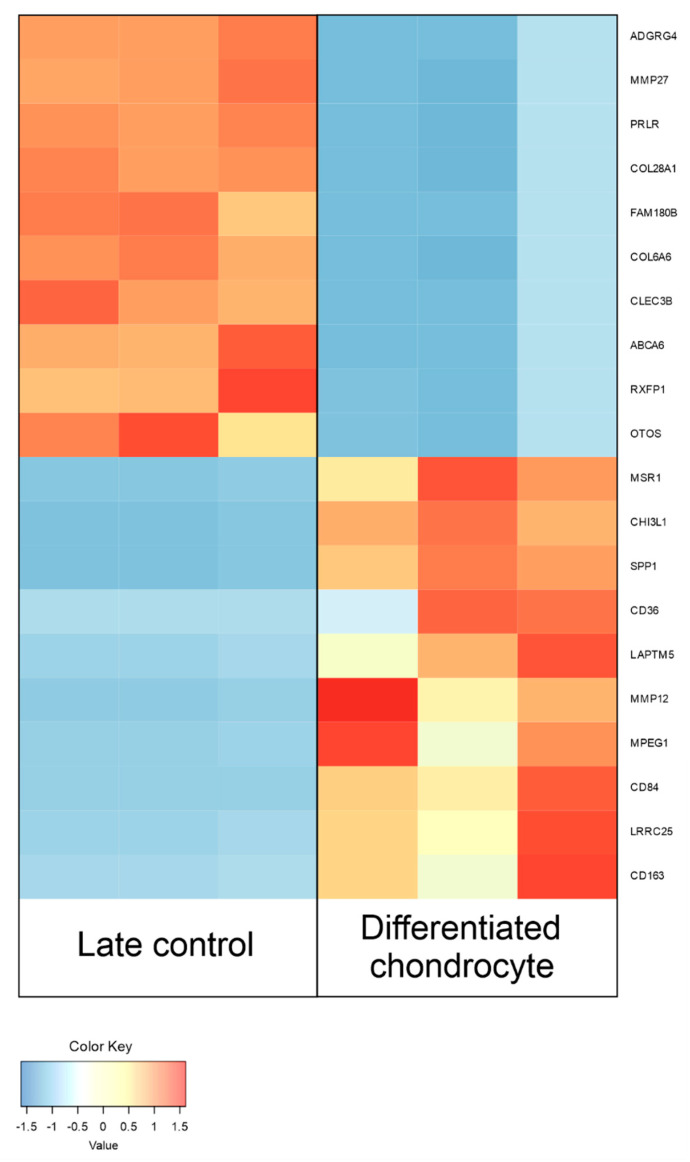
Heatmap presenting the changes in the 10 most up- and down-regulated genes between differentiated chondrocytes and day 30 primary cASC culture (late control), presented as log2FC.

Between differentiated chondrocytes and early cultures (day 1), differential regulation of 11,602 genes, out of the total 19,335 detected during the analysis. In turn, in the differentiated chondrocyte vs. late culture control (day 30) comparison, similar characteristics were presented by 11,274 genes. Out of these sets, 10 most up- and down-regulated genes in each comparison were selected and presented on Table 2 and Table 3, and Figure 6 and Figure 7. While the expression of a significant majority of the significantly regulated genes was lowered after long-term culture and differentiation, there is also a large amount of genes in which a notable increase in expression (sometimes higher than 10-fold) was observed. These results stand in accordance with previous findings of our group based on similar transcriptomic methods, confirming their validity for further analysis [22,31].

**Figure 8:**
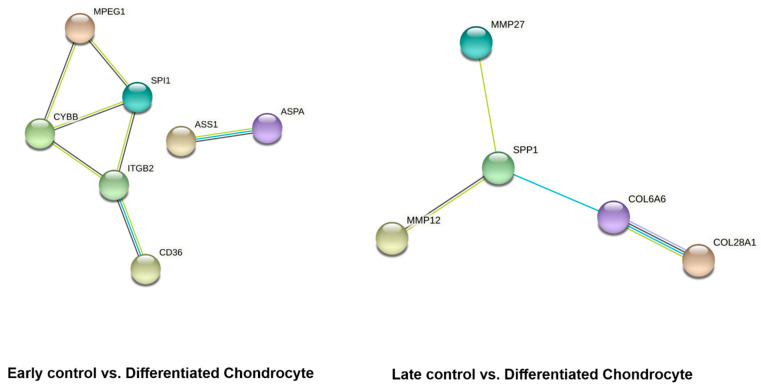
STRING analysis results visualising predicted interactions between the protein products encoded by the differentially expressed genes of interest. The genes not involved in any interactions were excluded from the figure.

In the last stage of bioinformatical analysis, the lists of differentially expressed genes of interest were uploaded to the STRING database to visualize the predicted interactions between their protein products. The results of the analysis are presented on Figure 8.

Out of the 10 most up- and down-regulated genes, seven showed predicted interaction in early control vs. differentiated chondrocyte comparison, and five in late control vs. differentiated chondrocyte. While the number of predicted interactions between the genes of interest is relatively low, the results of the STRING analysis were filtered based on the “high-confidence prediction” criteria, to ensure the maximum value of the results obtained from this in silico analysis. Furthermore, it cannot be out ruled that some of the genes not included in the results present interactions via other genes that were not detected to be differentially expressed in this study.

### 3.5. RT-qPCR Validation

**Figure 9:**
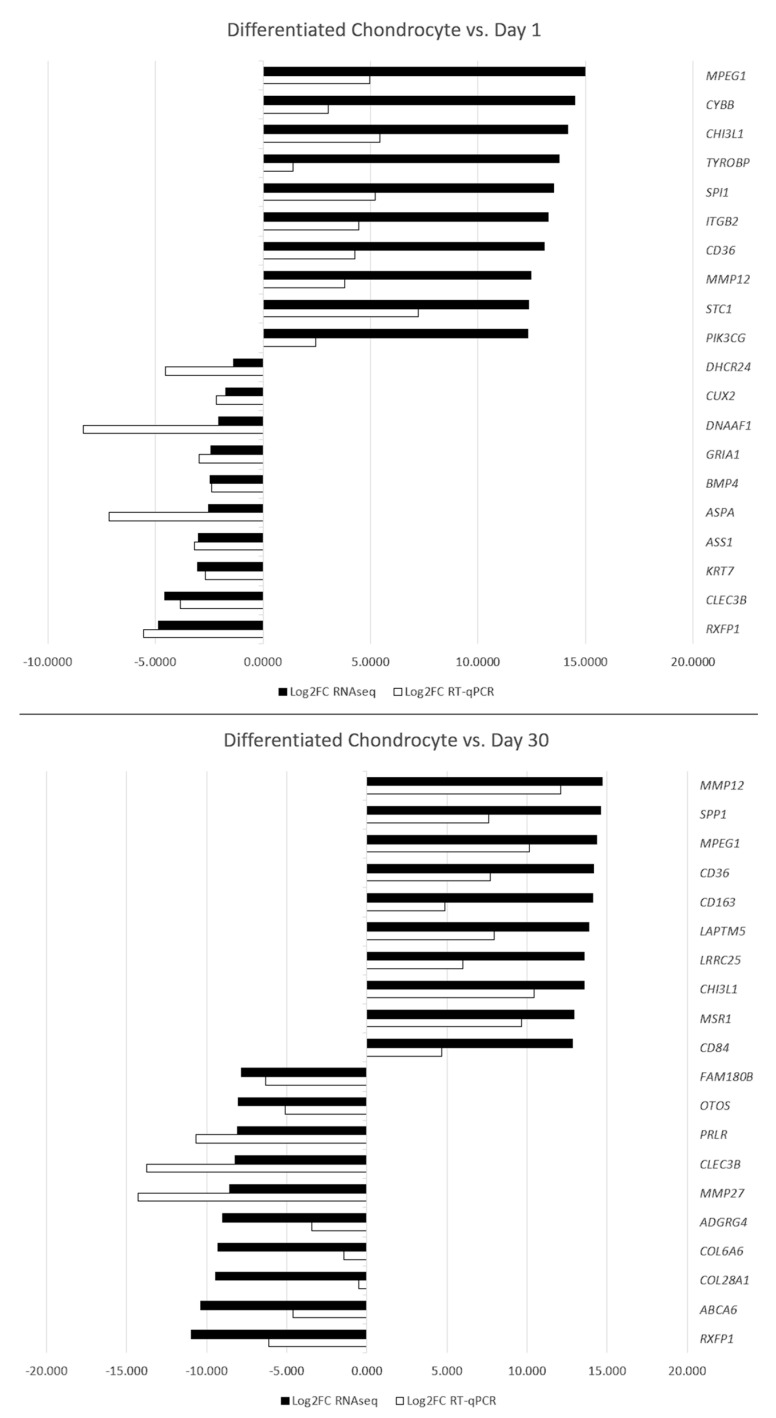
RT-qPCR validation results presented as log2FC.

To confirm the results of the RNAseq analysis, RT-qPCR was performed using specific primers. The results were presented and compared in the form of Figure 9.

The results of the RT-qPCR validation confirm the expression changes of genes between the analyzed groups. While there is some variation in the scale of change, it most likely results with a slightly different sensitivity of both methods and does not significantly impact the findings.

## 4. Discussion

A transcriptomic analysis of canine ASCs during their differentiation towards chondrogenic lineage is an important endeavor to identify a new molecular markers of ASCs differentiation [17]. It is especially important to identify differentiation markers in the context of studies stating that ex vivo conditions could significantly influence the stemness of ASCs, resulting in potentially malignant side effects of their therapeutic application [32]. Nonetheless, in vitro cultures remain a powerful tool of stem cell propagation and differentiation and will most continue to play an important role in further stem cell-related research and treatments. Hence, the transcriptomic assays can be related to the reports advocating for differentiation of stem cells before their potential clinical use, as the effectiveness and safety of such approach remains controversial in the literature [14]. NGS methods such as RNAseq are an especially useful transcriptomic tool, as they allow for wide scale analysis of a relatively small amount of nucleic acid material, often yielding a large amount of results that serve as an extensive reference for further research and clinical studies regarding particular genetic characteristics, such as disease-related mutations or polymorphisms [33]. Moreover, bioinformatical analysis of these results allows to evaluate the changes in the of gene expression profiles between the study and control groups, resulting in a number of differentially expressed biomarker candidates [31].

Differentiated chondrocytes were firstly related to the early culture control. The ten most upregulated genes between those groups included three that were previously directly associated with chondrocyte-differentiation related processes. Among them, the expression of *MPEG1* was previously described to be increased with the proceeding time of 3D chondrocyte culture, which corresponds with our findings, as it was significantly higher in chondrocytes compared to both controls [34]. A similar role can be attributed to *CH13L1*, which encodes a cartilage associated protein, and has been previously described in both normal and osteoarthritic subjects [35,36]. *ITGβ2* was also implicated in chondrogenic differentiation, with its crucial role in this process confirmed by knockout studies [37]. Furthermore, *CD36* is described in the literature as a marker of chondrocyte hypertrophy associated with response to inflammatory stimuli, which could be supported by the results of this study, as ex vivo differentiation is often associated with inflammation-like stimulus affecting the studied cells These four genes can therefore be considered as markers of in vitro differentiated chondrocytes, both due to their literature-reported functions and significant upregulation compared to undifferentiated ASC cultures.

While the further three genes were described in the context of chondrocytes, the literature does not report on their direct involvement in their in vitro culture and differentiation. [38]. Nonetheless, the expression of *MMP-12* was previously detected to be induced in chondrocytes during fetal and malignant development, which could indicate that the process if in vitro differentiation is associated with increased plasticity, which might have potential implications in the potential application of this process in clinical practice [39]. While we have detected *STC1* to be upregulated, it was previously associated with suppression of chondrogenesis [40]. Furthermore, the protein encoded by *STC1* was also implicated in the transport of calcium and phosphate transport, the processes related to cell metabolism and calcium/phosphate homeostasis [40]. It may be necessary to re-evaluate the role of in the STC1 differentiation process, as the results of our analysis are contrary to those available in the literature. A similar role could be attributed to *PIK3CG,* as the protein product of these gene was previously identified as a factor playing a role in cartilage destruction, indicating the potential need for the turnover of damaged or abnormal cells in culture. Moreover, this gene is a known modulator of extracellular signal and plays a role in E-cadherin mediated cellular adhesion, which might indicate its upregulation might also be related to the increase in the mass of cultured spheroids [41]. Overall, while these genes could be related to the process of chondrogenesis, they are most likely associated with the response of the culture to the ex vivo conditions, rather than differentiation. Hence, due to their significant upregulations in the studied cells, they could be considered as new markers of in vitro conditions’ influence on the differentiating ASCs.

The remaining three genes that were upregulated between differentiated chondrocytes and 1-day-cultured ASCs were not previously described with any chondrocyte- or stem cell-related literature. *CYBB* encodes a component of NADPH oxidase, which is responsible for free radical production, playing a role in response to microbial infections, which might implicate its increase in the cellular stress associated with in vitro conditions [42]. While *TYROBP* encodes a protein that plays key roles in immune systems, signal transduction and osteoclast activity, the exact role of its activation in the context of chondrocyte differentiation remains unclear [43]. Similarly, *SPI1* is a proto-oncogene with roles in immune system dysregulation, but its role in the context of in vitro chondrogenesis cannot be confidently suggested based on the available literature [44]. While the above-mentioned genes were not previously associated with any chondrogenesis- or ASC-associated processes, their significant upregulation in our study suggest them as potential candidates for new gene markers of their in vitro differentiation and/or long-term culture.

Among the 10 most downregulated genes the early control and differentiated chondrocytes five were previously associated with stem cell related processes. *DHCR24* encodes a potent reactive oxygen species (ROS) scavenger, protecting chondrocytes from ROS related damage, with its knockout resulting in an absence of chondrocyte proliferation [45]. Its significant reduction in our culture might be a signal of the negative impact of long-term in vitro culture on the identity of the finally differentiated chondrocytes, potentially advocating against the use of such cultures in a clinical setting. In turn, while a number of studies indicated *BMP4* as a crucial factor in chondrogenic differentiation of ASCs, Shu et al. proved that its role is not as significant as that of BMP2, which seems to be supported by findings of our study [46,47,48]. Lower levels of *ASS1* were associated with lower number of aggregated cells in chondrocyte differentiated MSCs, which finds confirmation in our study as the final chondrocyte spheroids were significantly smaller than the initial 3D cultured ASCs [49]. *KRT7* upregulation in MSC cultures was associated with exposure to tumor-associated factors and progression towards carcinoma-associated fibroblast-like phenotype [50]. Hence, the low levels of this gene in the cultures possibly occurred due to the induced differentiation towards chondrocytes, which mitigated the possibility of ASC progression towards fibroblast-like lineage. *CLEC3B* was detected to be decreased in degraded chondrocytes affected with osteoarthritis, which might indicate low viability of their long-term in vitro differenced cultures [51]. While significant downregulation of *KRT7* and *CLEC3B* were not previously associated with chondrogenic differentiation, our results suggest that it might be an important component of this process. Furthermore, it is important to reevaluate the role of *BMP4* in chondrogenesis, as successful ASC differentiation occurred despite its notable downregulation. The downregulation of the rest of the genes seems to indicate that while the differentiation process was successful, in vivo, and clinical studies need to be mindful of the potentially lower viability and possibly malignant tendencies of the in vitro cultured cells.

The remaining five genes that were downregulated between differentiated chondrocytes and 1-day-cultured ASCs were not previously described with any chondrocyte- or stem cell-related literature. *CUX2* encodes a gene predominantly detected in nervous tissues, with a significant downregulation of its expression possibly associated with mitigation of neurogenic potential of ASCs through induced chondrogenic differentiation [52]. *DNAAF1* is a gene involved in in ciliary transport, with singular reports of its implication in pathologies such as neural tube defects. However, none of the available literature allows to connect it to the context of ASC in vitro differentiation into chondrocytes [53,54]. A similar occurrence can be observed regarding *GRIA1*, which has mostly associated with susceptibility to mental disorder and cancer [55,56,57]. The aspartocyclase protein encoded by *ASPA*, is mostly detected in the central nervous system, which might again implicate the decrease in its expression in the loss of ASC neurogenic potential resulting from induced chondrogenic differentiation [58]. Finally, the roles of *RXFP1* have recently begun to be elucidated, but this gene’s expression has not previously associated with any MSC, or chondrocyte associated topics, which makes the role or effect of its downregulation unclear. Nonetheless, participation of the protein encoded by these genes has previously been implicated in topics such as cancer and fibrosis [59]. While none from these genes have previously been associated with any topics related to chondrogenesis or ASCs, their significant downregulation could be implicated in the differentiation or long-term culture process, suggesting them as potential negative markers.

The comparison between differentiated chondrocyte and 30-day undifferentiated control, account for the possible transcriptomic differences resulting from the conditions of 3D in vitro culture rather than chondrogenic differentiation itself. The 10 most upregulated genes in this group included nine that were associated with the topic of stem cells in the literature. *MMP12*, *MPEG1*, *CHI3L1,* and *CD36* were already described in the first comparison, as they were significantly upregulated between differentiated chondrocytes and both control groups, emphasizing their role as markers of ASC chondrogenic differentiation. Moreover, while *SPP1,* encoding osteopontin, was previously indicated as a bone-related marker, our results indicate that a notable increase of its expression could possibly also associated with chondrogenic differentiation of ASCs [60].The protein encoded by this gene was proven to serve roles in a variety of processes, from apoptosis and biomineralization to cell activation, immune response, and malignant progression. Hence, it cannot be ruled out that while it is not usually detected in physiological chondrocytes, it might play a role in their ex vivo differentiation. These five genes seem to be most significantly associated with the in vitro chondrogenic differentiation process, and hence should be taken into consideration as its new markers.

*CD163* was associated with a phagocytic phenotype of some chondrocytes, implicated in the processes of degraded tissue elimination [61]. Upregulation of this gene in chondrocyte spheroids could be related to the need for elimination of cells damaged by in vitro conditions and the chondrogenic medium, as the viability of ASCs subjected to differentiation was undoubtedly lowered, which reflects in the difference in size between control and differentiated spheroids. upregulation of *LAPTM5* and *MSR1* during long-term in vitro chondrogenic differentiation of MSCs was also previously reported by James et al. [34]. *LRRC25* does not yet have a reported role in stem cell biology. While its significant upregulation during long-term chondrocyte differentiation might indicate it as a potential new marker of this process, Du et al. implicated it in the pathways associated with innate pathogen response, so its expression might be associated with 3D culture stress [62]. The final upregulated gene in this comparison was *CD84*, which was reported to be upregulated in embryonic pre-chondrocytes vs. immature resting fetal chondrocytes in a study of Wu et al. [63]. This fact might indicate that this gene’s expression is more characteristic for chondrogenic lineage cells of higher plasticity, which could be an important factor in the context of differentiated chondrocyte application in clinical practice. Overall, while these five genes have a strong potential to be considered as markers of the in vitro chondrocyte differentiation process, mostly due to their significant upregulation in relation to ASCs cultured without chondrogenic medium, further studies are needed to fully rule out the possibility of the change of their expression due to other culture conditions-associated factors.

Among the 10 most downregulated genes in the differentiated chondrocyte compared to 30-day control, *CLEC3B* and *RXFP1* have the biggest potential to serve as a marker of the in vitro ASC differentiation process, as their expression was also significantly altered in chondrocytes vs. day 1 control. Among the remaining genes, six were previously associated with the topic of stem cells. Down-regulation of *PRLR*, a gene encoding the prolactin receptor, was previously reported in differentiating chondrocytes, decreasing their susceptibility to prolactin and increasing their viability [64,65]. Surprisingly, *MMP27* was reported to be upregulated in chondrocytes compared to nucleus pulposus cells, indicating it as a potential marker of this lineage [66]. Downregulation of MMP27suggests that while the baseline characteristics and expression of some markers confirms the identity of the in vitro differentiated chondrocytes. However, they possibly exhibit some characteristics different than their physiological counterpart, which might have implications in their potential clinical application. Among the two significantly downregulated genes encoding different collagen subunits, *COL28A1* presence was previously associated with MSC extracellular matrix production facilitating cell spreading, while lowered levels of *COL6A6* were characteristic for MSC chondrogenic differentiation [67,68]. Downregulation of both of these genes likely occurred due to the characteristics of 3D chondrogenic culture and could be considered a characteristic of this model system. *ABCA6* was previously detected to be upregulated in MSCs, making its downregulation likely associated with the change of their identity towards chondrocytes [69]. The last four genes from this comparison were not previously associated with any stem cell-related processes. The *OTOS* gene has only been associated with the development of inner ear structures [70]. While the role or reasons for its downregulation during chondrogenic differentiation of ASCs remain unclear, it is the first instance in which such an occurrence has been reported. *ADGRG4* encodes an adhesion G protein-coupled receptor, potentially suggesting higher activity of G-protein receptor pathways in MSCs than differentiated chondrocytes [71]. Finally, while information on *FAM180B* remain sparse, its significant downregulation detected in our study might indicate its role in processes associated with MSCs [72]. Overall, while downregulation of some of the genes of this groups has the potential to be considered as a marker of the in vitro chondrogenic differentiation process, the literature sources suggest that decreased expression of some is likely associated with the side effects of ex vivo induced chondrogenesis. Nonetheless, the knowledge on several of these genes remains sparse, suggesting that their downregulation could potentially play a role in ASC differentiation.

In conclusion, there are several genes identified in this study, such as *MMP12, MPEG1, CHI3L1*, and *CD36,* which show a strong potential to be considered as new markers of the in vitro ASC chondrogenic differentiation process. Furthermore, significant downregulation of genes, such as *CLEC3B* and *RXFP* showed a significant downregulation in both the studied comparisons, suggesting that the lowering of their expression could be characteristic for in vitro chondrogenesis. As for the remaining genes, while some of them showed differential expression, more studies are needed to rule out the possibility that the change of their expression did not occur due to other factors, unassociated with the process of differentiation itself. Moreover, significant regulation of several genes associated with increased plasticity and malignancy in the literature highlights the need for further in vivo studies to fully confirm the safety of differentiated ASC application in a clinical setting. Finally, differential regulation of several genes that are still poorly described in the literature e.g., *OTOS* and *FAM180B* suggests their role in long-term in vitro culture and chondrogenic differentiation of ASCs, as well as previously undescribed properties. Overall, while the results of our study provide an interesting insight into the functioning of in vitro cultured and differentiated ADSCs, strongly suggesting some of the genes of interest as potential new markers of long-term culture and chondrogenic differentiation-associated processes, further in vivo and clinical studies are needed to fully confirm the proposed functions, as well as to potentially apply the results of our study in potential therapeutic approaches in veterinary medicine. Furthermore, the potential future translation of the study results into knowledge regarding other models requires further bioinformatical analysis comparing them with the respective mouse or human datasets. Nonetheless, our findings prove the value of extensive molecular research into the molecular mechanisms governing ASC physiology.

## Figures and Tables

**Table 1:** The primer sequences used in this study and Ensembl IDs of the genes used for their design.

GENE NAME	FORWARD PRIMER	REVERSE PRIMER	ENSEMBL ID
***ABCA6***	GGTCAACTTCCTGGGCTACT	TTCGCCTGAACATTGAGCTG	ENSCAFG00845005313
***ACTB***	TCGAGACTTTCAACACCCCA	CATGAGGTAGTCGGTCAGGT	ENSCAFG00030015381
***ADGRG4***	TACAGCCTTGACTCTTGGGG	CCAGACTCAGAGGCCTTGAA	ENSCAFG00000018940
***ASPA***	CAAGGGGTTCTGAGAGCTGA	GCGGTTTCCAGTCTTGATCC	ENSCAFG00000019330
***ASS1***	GTGTGAATTTGTCCGCCACT	TCTGGAGGCGGTGATATTCC	ENSCAFG00030010628
***BMP4***	GAGAAGCAGCCAAACTACGG	CTTATTCTTCTTGCGGGCCC	ENSCAFG00030003207
***CD163***	CTCTGCAACTCTCACTGGGA	CAATCTCCCATGTGCTGCTC	ENSCAFG00030019149
***CD36***	CAGGAAGTGGTTGCGAACAG	AGCCAGATTGAGAACGGTCA	ENSCAFG00030010230
***CD84***	AACATACAGCTGGAGTCCCC	ACAGAGAGAGCATGACCAGC	ENSCAFG00000012569
***CHI3L1***	CACGTCATCTACAGCTTCGC	ACCAAAGCTCCATCCTCCAA	ENSCAFG00030003216
***CLEC3B***	AAATGCTTCCTGGCCTTCAC	CTCCGTCTCCCAGTTCTTGT	ENSCAFG00000014049
***COL28A1***	GGGACAAGGGAGATTTGGGA	GTCTGGCCTACTTCACCCTT	ENSCAFG00000029367
***COL6A6***	CTTCCGGGAGAGATGGGATC	TTCATGCGCTCGAATTCCTG	ENSCAFG00000006035
***CUX2***	GGGACCCAAGATGAACCAGA	CCGCTTCTTCTTCTGCATCC	ENSCAFG00000008575
***CYBB***	CTGAGCGAATTGTACGTGGG	GAGATCGCCAAAACCGTACC	ENSCAFG00030009532
***DHCR24***	AAGCAGGTACGGGAATGGAA	GTCCACCTCCAGAATGTCCA	ENSCAFG00040000097
***DNAAF1***	ACCCAAGCAAGCAGAAACAG	TCCAGCCAGAGACAACGTAG	ENSCAFG00000019964
***FAM180B***	AGTTCCAGGACCTGCGTAAA	GGGAAAGGGGTCAAGGATCA	ENSCAFG00000032106
***GRIA1***	TGACATTTCTCCCAGGTCCC	CTAGGTCCTCAGCACTCTCG	ENSCAFG00030007058
***HPRT1***	CCCAGCGTCGTGATTAGTGA	AGAGGGCTACGATGTGATGG	ENSCAFG00030008563
***ITGB2***	ATCAACGTCCCGATCACCTT	TCGCAGTTCTTCCCGATGTA	ENSCAFG00000011039
***KRT7***	TTCGCCTCCTTCATCGACAA	CGAAGATGCTGGGGAGGC	ENSCAFG00030022127
***LAPTM5***	TCCAAGGTCCCACTGATGAC	TCCACACGCACTTGAACATG	ENSCAFG00030004981
***LRRC25***	TGGTTCTAGGTCTGTGGCTG	GGGCCCTCGTAGTTCATGTA	ENSCAFG00000014879
***MMP12***	AGATTCTTGTGGTGGAGGCA	TGGCTGTGGTCTCAAATTGC	ENSCAFG00030013517
***MMP27***	TTCCCAAACCCATCCGTACA	CTGGAAAGCAGCATCGACTC	ENSCAFG00000015066
***MPEG1***	GAGGTCAAGGGAGAAGGGAC	GGGTGCACACGTGTATGATC	ENSCAFG00000007649
***MSR1***	ATGCTCGTTCAATGACAGCC	GTGCTGCCATGATTCCGATG	ENSCAFG00030021708
***OTOS***	CCTACTGGCCTTTCTCCACT	CTGCTGATAGGGGACATGGA	ENSCAFG00000031916
***PIK3CG***	AGGCAGCTGTGGAGAGATTT	AGGAAGTCTGGGGTTAGCAC	ENSCAFG00030020245
***PRLR***	TGGGCAGCAGACTCAGTTTA	ATGACAGCAGAAAGAACGGC	ENSCAFG00040010421
***RXFP1***	AGGTCTGAGAACAAGCTGCA	AGATCCCACAAGCTGACAGT	ENSCAFG00000008672
***SPI1***	GACTATCTCCCAGTGGCAGG	TTTGCACGCCTGTAACATCC	ENSCAFG00000008723
***SPP1***	TATTCACTCCAGCTGTCCCC	TGTCTTTTGCATGGCTGTCC	ENSCAFG00000009569
***STC1***	TTCTGTGAGCCCCAGGAAAT	CAGCGCTGTACAAGAAGGAT	ENSCAFG00000009104
***TYROBP***	CAACTGCCCCGTGGTGAG	GATGCGCTGTTTCCTGGTC	ENSCAFG00030007988

**Table 2:** Differentially expressed gene of interest list between differentiated chondrocytes and day 1 culture control. FC—fold change, adj. *p* value—adjusted *p* value.

Differentiated Chondrocytes vs. Day 1			
Gene Symbol	Log_2_FC	adj. *p* Value	Entrez Gene ID
MPEG1	14.9622	9.90 × 10^3^	475960
CYBB	14.4821	4.24 × 10^2^	491825
CHI3L1	14.1706	9.09 × 10^6^	490222
TYROBP	13.7521	5.66 × 10^4^	476477
SPI1	13.5224	1.59 × 10^3^	611255
ITGB2	13.2486	1.11 × 10^2^	403770
CD36	13.0788	4.36 × 10^2^	475931
MMP12	12.4532	2.41 × 10^3^	611789
STC1	12.3310	1.92 × 10^2^	486112
PIK3CG	12.3146	6.00 × 10^3^	483266
DHCR24	−1.3651	1.65 × 10^4^	489573
CUX2	−1.7343	3.13 × 10^4^	486267
DNAAF1	−2.0651	4.95 × 10^2^	479628
GRIA1	−2.4020	2.51 × 10^2^	489168
BMP4	−2.4524	6.73 × 10^3^	490695
ASPA	−2.5116	2.23 × 10^2^	611064
ASS1	−2.9978	1.37 × 10^2^	480693
KRT7	−3.0224	2.13 × 10^2^	477602
CLEC3B	−4.5737	2.69 × 10^3^	609596
RXFP1	−4.8416	1.36 × 10^2^	100855494

**Table 3:** Differentially expressed gene of interest list between differentiated chondrocytes and day 30 culture control. FC—fold change, adj. *p* value—adjusted *p* value.

Differentiated Chondrocyte vs. Day 30			
Gene Symbol	Log_2_FC	adj. *p* Value	Entrez Gene ID
MMP12	14.673	2.36 × 10^3^	611789
SPP1	14.597	4.75 × 10^5^	478471
MPEG1	14.369	9.75 × 10^3^	475960
CD36	14.138	4.26 × 10^2^	475931
CD163	14.096	2.81 × 10^2^	477704
LAPTM5	13.879	1.58 × 10^2^	487324
LRRC25	13.590	1.83 × 10^2^	609889
CHI3L1	13.562	6.28 × 10^6^	490222
MSR1	12.912	6.36 × 10^4^	482891
CD84	12.843	6.40 × 10^3^	488641
FAM180B	−7.843	1.89 × 10^5^	100685781
OTOS	−8.031	2.57 × 10^4^	477428
PRLR	−8.092	1.70 × 10^6^	479363
CLEC3B	−8.239	2.67 × 10^5^	609596
MMP27	−8.569	6.44 × 10^6^	489430
ADGRG4	−8.987	3.93 × 10^6^	492163
COL6A6	−9.286	2.61 × 10^6^	610649
COL28A1	−9.442	7.12 × 10^7^	482315
ABCA6	−10.357	4.77 × 10^5^	480456
RXFP1	−10.948	1.65 × 10^4^	100855494

## Data Availability

All of the data discussed in this work, if not already included in the manuscript, are available from the corresponding author on reasonable request.
